# Safety of Cinnamon: An Umbrella Review of Meta-Analyses and Systematic Reviews of Randomized Clinical Trials

**DOI:** 10.3389/fphar.2021.790901

**Published:** 2022-01-18

**Authors:** Dan-Tong Gu, Tao-Hsin Tung, Zhu Liduzi Jiesisibieke, Ching-Wen Chien, Wen-Yi Liu

**Affiliations:** ^1^ Institute of Otolaryngology, Clinical Research Center, Fudan University Affiliated Eye and ENT Hospital, Shanghai, China; ^2^ Evidence-based Medicine Center, Taizhou Hospital of Zhejiang Province Affiliated with Wenzhou Medical University, Linhai, China; ^3^ Institute for Hospital Management, Tsing Hua University, Shenzhen, China; ^4^ Department of Health Policy and Management, Bloomberg School of Public Health, Johns Hopkins University, Baltimore, MD, United States; ^5^ Shanghai Bluecross Medical Science Institute, Shanghai, China

**Keywords:** safety, cinnamon, umbrella review, systematic reviews, meta-analyses

## Abstract

**Purpose:** Many evidence-based studies have indicated that cinnamon has therapeutic effects. However, it may not be entirely safe and its adverse effects may be ignored. The present umbrella review was conducted to elucidate the safety of cinnamon.

**Methods:** Pertinent meta-analyses and systematic reviews of randomized controlled trials on cinnamon use in humans were identified by searching PubMed, EMBASE, and the Cochrane Library from their inception to September 15, 2021. All meta-analyses and systematic reviews on the safety or adverse effects of cinnamon were considered. PRISMA 2020 was used as the standard of reporting (PRISMA registration ID: 286746).

**Results:** We identified three meta-analyses and one systematic review that described the safety of cinnamon. The quality of the meta-analysis and systematic reviews was evaluated using “Assessing the Methodological Quality of Systematic Reviews.” Their quality was rated as low in two (50%) instances and moderate in two (50%). There were no significant toxic- or side effects between cinnamon group and placebo group regardless of dose and duration.

**Conclusion:** There is evidence to support that the use of cinnamon has no adverse reactions. It can improve the health status of patients as an adjuvant treatment. Future studies exploring better profile risks and protective factors for cinnamon use-related adverse effect are needed, in order that preventive approaches can be developed.

## 1 Introduction

Cinnamon obtained from *Cinnamomum* verum J. Presl (family Lauraceae) is a common spice used worldwide and a tropical medicine. It contains manganese, iron, dietary fiber, calcium, their derivatives, and other related compounds (Abraham et al., 2010). Cinnamon is a popular ingredient in cooking, medicine, forage, and is used in many industries (Michiels et al., 2007; Santos and da Silva, 2018). From a clinical viewpoint, it is often used in diabetes treatment because of its hypoglycemic and lipid-lowering potential (Santos and da Silva, 2018; Zare et al., 2019). It has also been found to be useful in reducing glycated hemoglobin (HbA1c) and fasting blood glucose levels in patients with type 2 diabetes (Pauline and Maddox, 2017). In addition, cinnamon has antimicrobial and antioxidant properties, and its application has been recommended singly or as a supplement in the treatment of cancers such as promyelocytic leukemia (Jayaprakasha and Rao, 2011; Assadollahi et al., 2013). Cinnamon bark, cinnamon twig, and shaved cinnamon bark differ in their compound compositions, and these differences could be used to achieve quality control when using cinnamon (Chen et al., 2016). Cinnamon bark contains natural antioxidants, that could reduce the risk of cancer, and signs of aging (Ghosh et al., 2015). Cinnamon twigs are commonly used for treating inflammatory diseases and amenorrhea in China (Ghosh et al., 2015). Shaved cinnamon bark together with other traditional Chinese medicines could delay the process of the deterioration of some heart diseases such as congestive heart failure (Huang, 2014).

Despite the several clinical benefits afforded by cinnamon, concerns about its safety persist (Kort and Lobo, 2014; Deyno et al., 2019). The results of some studies have indicated that the safety of cinnamon is related to parameters such as fasting blood glucose, serum insulin, and alanine aminotransferase levels (Blevins et al., 2007; Akilen et al., 2012; Shishehbor et al., 2018). With respect to adverse effects, while some studies reported no adverse effects in individuals treated with cinnamon, indicating the safety of this traditional medicine (Mang et al., 2006; Suppapitiporn et al., 2006), whereas others reported dermatological problems (Altschuler et al., 2007; Crawford, 2009). Studies of different dosages of cinnamon have mostly been conducted in animals. Thus, the evidence for the safety of cinnamon remains limited and controversial, and the potential safety problems remain unknown. The present umbrella review was conducted to elucidate the safety of cinnamon based on the existing systematic reviews and meta-analyses of randomized clinical trials (RCTs), which may facilitate a better understanding of the side effects of cinnamon among healthcare workers and policy makers.

## 2 Methods

### 2.1 Search Strategy and Eligibility Criteria

This umbrella review involved an evaluation of pertinent systematic reviews and meta-analyses, so as to draw more reliable conclusions (Smith et al., 2011; Aromataris, 2014). PubMed, EMBASE, Cochrane Library, and the Web of Science were systematically searched from their inception to September 15, 2021, to identify systematic reviews and meta-analyses of RCTs examining the safety of cinnamon. The search string used was “[(cinnamo* OR cinnamic) AND (safety OR security) AND (efficacy OR efficiency OR effect*) AND (hepa* OR liver)]” without a language restriction (Table 1). The protocol for this systematic review was recorded in PROSPERO with the identification number 286746.

**TABLE 1:** Search strategy until August 10, 2021.

		PubMed	Embase	Cochrane	Web of science
#1	Cinnamo*	6,537	3	523	13,913
#2	Cinnamic	28,049	9,996	45	8,123
#3	Safety	720,387	1,404,766	261,301	958,659
#4	Security	124,523	104,253	3,436	413,579
#5	Efficacy	942,083	1,868,531	385,327	1,128,079
#6	Efficiency	1,100,052	574,856	19,035	1,954,432
#7	Effect*	10,087,377	6,113,819	1,039,614	13,376,402
#8	Hepa*	1,083,168	3,792	69,829	961,359
#9	Liver	1,197,424	1,788,028	64,507	949,464
#10	#1 or #2	33,921	9,999	560	21,500
#11	#3 or #4	833,939	1,496,295	263,913	1,348,452
#12	#5 or #6 or #7	11,034,060	7,662,872	1,176,084	15,198,353
#13	#8 or #9	1,716,602	1,790,406	103,091	1,533,755
#14	#10 and #11	888	384	110	446
#15	#10 and #12	21,806	3,097	505	8,614
#16	#10 and #13	3,336	483	79	1,128
#17	#10 and #11 and #12	717	180	108	338
#18	#10 and #12 and #13	2,458	237	78	636
#19	#17 and #18	101	24	31	50

The exclusion criteria were as follows: 1) studies without safety evaluation; 2) studies on pharmacokinetics that involved *in vivo* experiments; and 3) animal studies. The search was not limited by the dosage of cinnamon or the length of treatment. Only meta-analyses and RCTs were considered. The entire selection process was conducted by Wen-Yi Liu and Zhu Liduzi Jiesisibieke independently, and any disagreements were settled by discussion with a third principal author, Tao-Hsin Tung.

### 2.2 Data Extraction and Quality Assessment

For each selected meta-analysis, we focused on the following items: level of comparison, random-effects summary, I^2^, and small-study effects, a. k.a.,/excess significance bias. The following information was obtained from the selected meta-analyses: author and year, outcome, number of patients, number of study types, and AMSTAR 2 (Assessing the Methodological Quality of Systematic Reviews) assessment scores.

### 2.3 Assessment of Methodological Quality

The methodological quality of the works included was evaluated using the AMSTAR 2 guidelines, which includes16 items that systematically score evidence-based medicine studies (Shea et al., 2009; Poole et al., 2017). It is not intended to provide an overall score based on the evaluation results of each item, as a high score may mask some very serious methodological deficiencies and provide a high-quality evaluation (Shea et al., 2017) (Table 2). AMSTAR 2 is considered a reliable and valid tool for evaluating the quality of systematic reviews and meta-analyses of interventional and observational research (Shea et al., 2009; Pieper et al., 2014; Poole et al., 2017). It includes ratings for the quality of academic studies, statistical analyses, and openness of meta-analyses. Regarding the rating items for the methodological quality of meta-analyses, the fixed-effects model for the summary estimate was downgraded compared to the random-effects model. This means that the random-effects model was considered the most suitable to be used for pooled estimates due to the heterogeneity in study samples, study designs, methods of cinnamon preparation, and duration. A single real effect size was not considered relevant to all selected studies.

**TABLE 2:** Characteristics of the included studies.

Reference	Outcome investigated	Patients	Selection as most comprehensive	RCTs included	Prospective studies included	Retrospective studies included	Study quality (AMSTAR rating)
Mousavi et al. (2021)	Alanine aminotransferase	236	✓	9	0	0	Low
	Aspartate aminotransferase	222	✓				
	Alkaline phosphatase	53					
Zhou et al. (2016)	Gastrointestinal symptoms	641	✓	11	0	0	Low
	Headache or/and dizziness	281					
	Feeling of numbness in the mouth and tongue	641					
	Palpitation	200					
	Adverse events	641					
Leach et al. (2012)	Adverse events	264		10	0	0	Moderate
	Fasting blood glucose level	304	✓				
	Postprandial blood glucose level	40	✓				
	Glycosylated hemoglobin A1c (HbA1c)	405	✓				
	Serum insulin	81					
	Insulin sensitivity (CHO/unit insulin)	48					
	Insulin sensitivity (HOMA-IR)	25					
Deyno et al. (2019)	Fasting blood glucose	1,098	✓	16	0	0	Moderate
	Insulin resistance (HOMA-IR)		✓				
	Insulin						
	HbA1c level						
	Low-density lipoprotein (LDL) level (mmol/L)						
	HDL level (mmol/L)						
	TC level (mmol/L)						
	TG level (mmol/L)						
	BMI (kg/m^2^)						

### 2.4 Assessment of Epidemiological Credibility

Relationships that had the highest evidence and no hints of major heterogeneity or bias were determined (Bellou et al., 2018). We considered persuading the relationships that met all the following criteria were persuasive: statistical significance per random-effects model at a *p*-value of <0.000001 with more than 1,000 cases, no high heterogeneity among selected studies (I^2^ < 50%), 95% prediction interval (excluding the null value), and no evidence of small-study effects and significant bias. Associations with more than 1,000 cases, a *p*-value of <0.000001, and most studies indicating a significant effect were viewed as highly recommended. The associations supported by more than 1,000 cases and significant effects at a *p*-value of <0.001 were graded as “recommended.” Nominally significant associations (*p* < 0.05) were considered weak evidence. Evidence obtained from fewer than 1,000 samples was graded as poor.

## 3 Results

**FIGURE 1:**
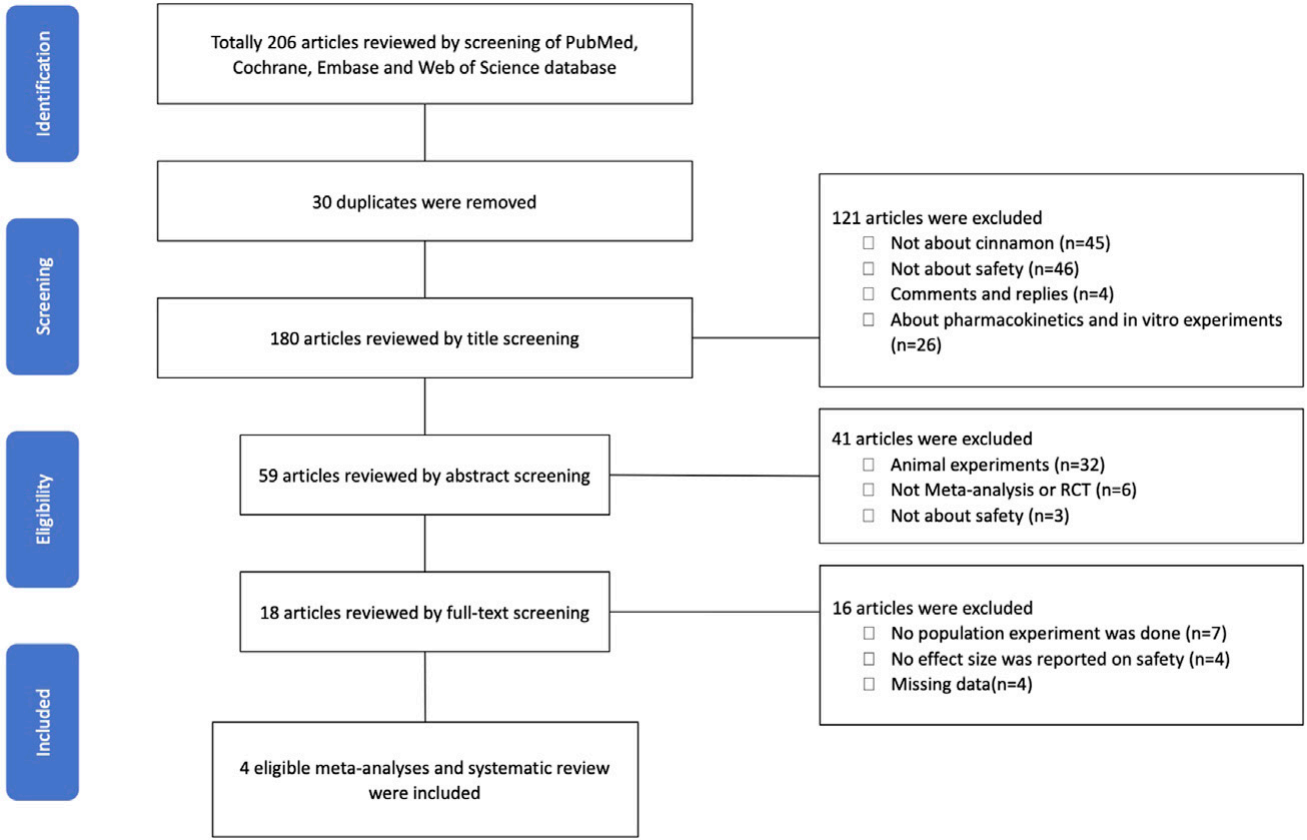
PRISMA flow diagram of the study.

A total of 206 articles and 180 articles were reviewed using title screening (Figure 1). We finally included four studies that fulfilled the eligibility criteria. Of these four studies, the study by Mousavi et al. (2021) included alanine aminotransferase, aspartate aminotransferase, and alkaline phosphatase levels as outcomes that would indicate the safety of cinnamon supplementation with respect to liver enzymes. The authors found that cinnamon improved serum levels of hepatic enzymes in patients with type 2 diabetes. The clinical benefits of cinnamon described Zhou et al. (2016) included gastrointestinal symptoms, headache and/or dizziness, sensation of numbness in the mouth and speech difficulties, palpitations, and adverse events as outcomes that would indicate the effectiveness and safety of the Shexiang Baoxin Pill (SXBXP, a patented Chinese medicine). The SXBXP is composed of Cortex Cinnamomic and other seven medical materials or extracts, and there was no significant difference in adverse events between the SXBXP and control groups in the study. Leach and Kumar (Leach and Kumar, 2012) included adverse events; fasting blood glucose, postprandial blood glucose, HbA1c, and serum insulin levels; and insulin sensitivity (CHO/unit insulin, homeostasis model assessment of insulin resistance [HOMA‐IR]) measured the efficacy and safety of cinnamon for the management of diabetes mellitus. The authors found no significant differences in adverse events, regardless of dosage and treatment duration, between the cinnamon and placebo groups. In a study by Deyno et al. (Deyno et al., 2019), no significant difference in safety was found between cinnamon and placebo. Furthermore, the authors concluded that cinnamon could help reduce the fasting blood glucose level and HOMA-IR values.

In terms of publication bias, Mousavi et al. (Mousavi et al., 2021) evaluated this bias using a funnel plot analysis and conducted Egger’s test. They found no small-study effects and no excessive significance bias. However, the number of trials included in some subgroups was relatively low. Zhou et al. (2016) did not conduct a funnel plot analysis or Egger’s test since less than 10 studies were included. In the study by Deyno et al. (2019), most of the included studies did not contain safety data, and the included studies had high heterogeneity. Thus, the results should be interpreted conservatively. Leach and Kumar, (2012) also did not conduct a funnel plot analysis. There were several explanations for asymmetry in their study, with publication bias being one of the possibilities.

## 4 Discussion

### 4.1 Pathogenesis

Cinnamic acid, syringic acid and choline are also included in the chemical composition of cinnamon, which includes volatile components, polysaccharides, sesquiterpenoids, and their glycosides and flavonoids. The main biological effects of cinnamon are derived from the phytochemicals themselves and their interactions.

### 4.2 Clinical Implications

To our knowledge, this is the first umbrella review of the safety of cinnamon. It has received attention not only for its health benefits but also for its potential adverse effects. In the three included studies, there was no significant difference in adverse events between cinnamon and placebo.

#### 4.2.1 Impact of Cinnamon on the Diabetes

Diabetes mellitus is concurrent with high morbidity and mortality and its prevalence is cumulative globally (Xiang et al., 2004). Cinnamon has been used as a supplement in the treatment of diabetes, cancer, and primary dysmenorrhea (Jahangirifar et al., 2018; Sadeghi et al., 2019; Careyva et al., 2020). A four-month double blinded, placebo-controlled trial using an aqueous extract of cinnamon in 22 subjects with impaired FBG reported a decline in fasting glucose as well as a decline in malondialdehyde, and plasma antioxidant markers were increased at the same time (Roussel et al., 2009). Cinnamon supplementation could improve glycemic management and alleviate oxidative stress in patients with diabetes and could thus decrease the release of liver enzymes (Allen et al., 2013; Deyno et al., 2019). Previous studies have shown that conventional antidiabetic agents have some side effects (Hanefeld, 2007; Inzucchi et al., 2012), whereas a study investigated the effect of combined polyherbal dietary supplement cinnamon, purple onion, and tea, and found that tea could lower lowering blood glucose. It was proved to be beneficial at the same time. (Weng et al., 2021). The possible hypoglycemic pathway exerts its action by enhancing the activities of bioenzymes such as hexokinase and pyruvate kinase during glycolysis by increasing the content of liver glycogen and inhibit the gene expression of glucose-6-phosphatase and phosphoenolpyruvate carboxylase, which are key enzymes in the process of liver gluconeogenesis, and to maintain insulin resistance (Prasath and Subramanian, 2011; Prasath et al., 2014).

#### 4.2.2 Impact of Cinnamon on Bacterial Infections and Tumor

Cinnamon is also a source of antibiotics, particularly in the context of multidrug-resistant bacterial infections (Vasconcelos et al., 2018). Nanoparticle-based cinnamon oil gel is very effective for the treatment of burn wound infection (Wen et al., 2021). The essential oils of cinnamon also exert potent antiviral effects against influenza type A virus (Wani et al., 2021). Cinnamon extracts, essential oils and their compounds have been proven to inhibit bacteria through damage to the cell membrane; alteration of the lipid profile; and inhibition of ATPases, cell division, membrane porins, motility, and biofilm formation; and via anti-quorum sensing effects (Vasconcelos et al., 2018). Cinnamon bark has also been proven effective in the treatment of methicillin-resistant Staphylococcus aureus (MRSA) (Zouhir et al., 2016). Cinnamon bark essential oil is may also apply in combinatory therapies so as to act on a par with synergistic interactions (Yang et al., 2017).

Schoene NW et al. (Schoene et al., 2005; Schoene et al., 2009) found that cinnamon total polyphenols can inhibit the proliferation of acute lymphoblastic leukemia cells. The possible mechanism is that two signaling proteins, p38MAPK and cyclin B1, are regulated to disrupt the phosphorylation/dephosphorylation of G2/M phase and impede the G2/M phase of the cell cycle. Assadollahi V et al. (Assadollahi et al., 2013) found that cinnamon polyphenols could inhibit the proliferation of the HL-60 cell line on the basis of Schoene NW’s study, indicating that the antitumor effect of the extract was correlated with concentration and time, and this process was associated with the fact that cinnamon water extract promoted tumor cell apoptosis and stopped G1 phase of the cell cycle. Other studies (Koppikar et al., 2010) found that cinnamon water extract could effectively inhibit the proliferation of cervical cancer SiHa cells and further induce the apoptosis of SiHa cells. The mechanism could be that it downregulates the expression of MMP-2 and Her-2 proteins, enhances intracellular calcium channel signals, eliminates the correlation of mitochondrial membrane potential, and thus inhibits the metastasis of malignant tumor cells.

#### 4.2.3 Potential Side Effects of Cinnamon

The aforementioned clinical benefits have contributed to cinnamon consumption; however, there is evidence that cinnamon may have adverse effects. Coumarin, one of the main components of cinnamon, has been indicated to have hepatotoxic, and carcinogenic effects (Brancheau et al., 2015; Mousavi et al., 2021). The dose and duration of cinnamon supplementation in a meta-analysis included in this review did not exceed the daily tolerable coumarin intake. Higher cinnamon intake may lead to hepatotoxicity (Brancheau et al., 2015). Behar et al. (2016) showed that the cinnamaldehyde in electronic cigarettes may interfere with homeostasis in the respiratory tract. Clouet et al. (2019) found that cinnamaldehyde may result in skin sensitization. The tolerable daily intake of cinnamon has been determined to be 0.1 mg/kg/day and employed in Europe to ensure safe use (Abraham et al., 2010). Hossein, (2013) found that stomatitis, perioral dermatitis, gingivitis, contact dermatitis, and other hypersensitivity reactions can occur after exposure to cinnamic acid, especially as the toxicity of benzyl cinnamate is higher. Allyl cinnamyl ester is also irritating to human skin. Cinnamaldehyde and cinnamol are more toxic than cinnamic acid. A mere 1% of cinnamaldehyde can cause mild hepatic cell edema in animal experiments, but few studies have been conducted on humans. Cinnamaldehyde and cinnamol are strong skin sensitizers that can easily cause contact dermatitis.

Numerous studies have reported the advantages of cinnamon when used safely (Ghosh et al., 2015; Pauline and Maddox, 2017; Santos and da Silva, 2018; Zare et al., 2019), for example, in the treatment of diabetes and as a natural antioxidant in foods. Furthermore, the active ingredients of cinnamon have been proven helpful in controlling and preventing the complications of coronavirus disease 2019 (Prasanth et al., 2021; Zareie et al., 2021). Cinnamon provided whole or as an aqueous extract contains a different number of active agents with various antihyperglycemic actions, since hypoglycemic activity appears in both aqueous extract, and powdered bark (Anderson et al., 2004; Verspohl et al., 2005; Kim et al., 2006).

The results of umbrella systematic review indicated that cinnamon intake within the daily intake range did not have significant adverse effects. However, it is essential to discuss that while we evaluated the safety of cinnamon supplementation, it was not quantitatively analyzed in this review. Due to a lack of strict rules, the nutraceutical manufacturers must check the safety of a marketed good products that is applied at a lower dosage than in the pharmaceutical setting. It should be noted that systematic reviews and meta-analyses are at the top of the hierarchy of clinical practice.

### 4.3 Methodological Considerations

The main strength of the present umbrella systematic review is that it included systematic reviews, meta-analyses, and an evaluation of the overall evidence. However, there are some limitations of this work. First, the sample count was relatively low; hence, more in-depth studies involving larger samples are needed in the future. Second, the number of relevant meta-analyses and systematic reviews was relatively small; therefore, further investigation into the safety of cinnamon is necessary. Third, a study of the different dosages and durations of treatment is necessary. Fourth, the included systematic reviews or meta-analyses were of relatively low methodologic quality, thus reducing the significance, and reliability of the clinical evidence for cinnamon safety in this umbrella review. For example, there was no comparator or placebo; therefore, placebo evidences could not be entirely excluded. Finally, due to a lack of comprehensive information about the above safety profile, it is difficult to assess the effects of cinnamon on other biomarkers relevant to safety. This needs to be explored in future studies as well.

## 5 Conclusion

This study summarized existing evidence on the safety of cinnamon, showing that cinnamon dose not cause obviously increased adverse effects when used on a large scale. It also has benefits in the treatment of a variety of diseases, such as type 2 diabetes and cancer. In other words, while cinnamon is effective for many of its benefits, it does not increase the risk of injury or mortality. The results of this study implied that cinnamon can be used as an adjunctive drug in the clinic field in future years, and its safety can be guaranteed.

## Abbreviations

Hba1c, glycated hemoglobin; RCT, randomized clinical trial; AMSTAR 2, Assessing the Methodological Quality of Systematic Reviews; SXBXP, Shexiang Baoxin Pill; HOMA-IR, homeostasis model assessment of insulin resistance.

## Data Availability

The study data and materials are in the custody of the corresponding author and can be made available on reasonable request.

## Abbreviations

Hba1c, glycated hemoglobin; RCT, randomized clinical trial; AMSTAR 2, Assessing the Methodological Quality of Systematic Reviews; SXBXP, Shexiang Baoxin Pill; HOMA-IR, homeostasis model assessment of insulin resistance.
